# In tribute to Francesco Di Virgilio, a great scientist and a wonderful friend

**DOI:** 10.1007/s11302-026-10135-9

**Published:** 2026-02-12

**Authors:** Simonetta Falzoni, Anna Lisa Giuliani, Elena Adinolfi, Robson Coutinho-Silva, Peter Illes, Simon C. Robson, Yong Tang, Henning Ulrich, Charles Kennedy

**Affiliations:** 1 Dept. of Medical Sciences, University of Ferrara, Ferrara, Italy; 2 Laboratory of Immunophysiology, Biophysics Institute Carlos Chagas Filho, Federal University of Rio de Janeiro, Rio de Janeiro, Brazil; 3 Rudolf Boehm Institute for Pharmacology and Toxicology, University of Leipzig, 04107 Leipzig, Germany; 4 International Research Centre On Purinergic Signalling, School of Health and Rehabilitation, Chengdu University of Traditional Chinese Medicine, Chengdu, 611137 China; 5 Center for Inflammation Research, BIDMC, Harvard Medical School, 330 Brookline Avenue, Boston, MA USA; 6 Department of Biochemistry, Institute of Chemistry, University of São Paulo, São Paulo, Brazil; 7 Strathclyde Institute of Pharmacy & Biomedical Sciences, University of Strathclyde, 161 Cathedral Street, Glasgow, G4 0RE UK

**Keywords:** Francesco di Virgilio, P2X7 receptor, Inflammation, Cancer

## Abstract

Francesco Di Virgilio, one of the giants of purinergic signalling, died suddenly on September 22, 2024, which is an immense loss to so many friends and colleagues and especially to his beloved wife, Dorianna. Here, 1 year on, we pay tribute to him and his immense contribution to our field. Francesco graduated in medicine from the University of Padova, Italy, in 1979, with what became a lifelong interest in inflammation. He then held post-doctoral positions at University College London, Padova University, and Columbia University, New York, where he became acquainted with P2X7 receptors. He then returned to Padova as an Associate Professor of Molecular Pathology before moving to the University of Ferrara in 1992, where he set up a world-leading lab that studied the roles and underlying cellular mechanisms of the action of P2X7 receptors in inflammatory pathologies. Francesco published over 370 peer-reviewed articles, which have been cited > 35,000 times, giving him an H-index > 100. In addition, he filed several patents related to purinergic signalling. He also collaborated extensively, both within the University of Ferrara and worldwide, including in universities in the UK, Spain, Germany, the USA, and Brazil. Francesco was a man of great passion and intellect, who possessed scientific vision, intuition, and integrity, and he became the go-to world expert on P2X7 receptors and inflammation. We have lost a giant in the field and a dear friend, but he leaves behind an exceptional body of work, an outstanding legacy, and many friends who will miss him.

## Introduction

**Fig. 1:**
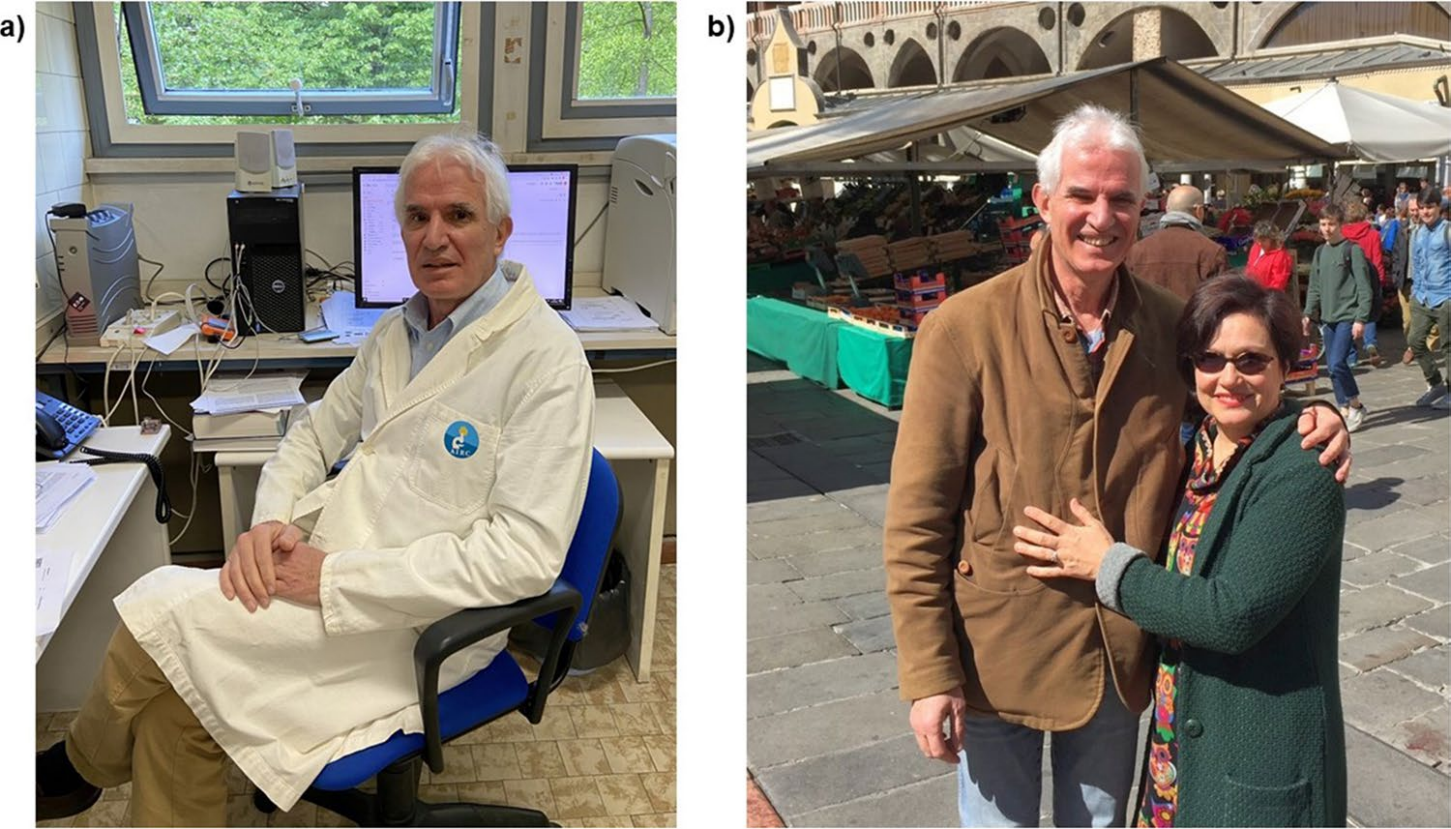
Francesco Di Virgilio. **a** In his office in 2021. **b** With Dorianna in 2015

**Fig. 2:**
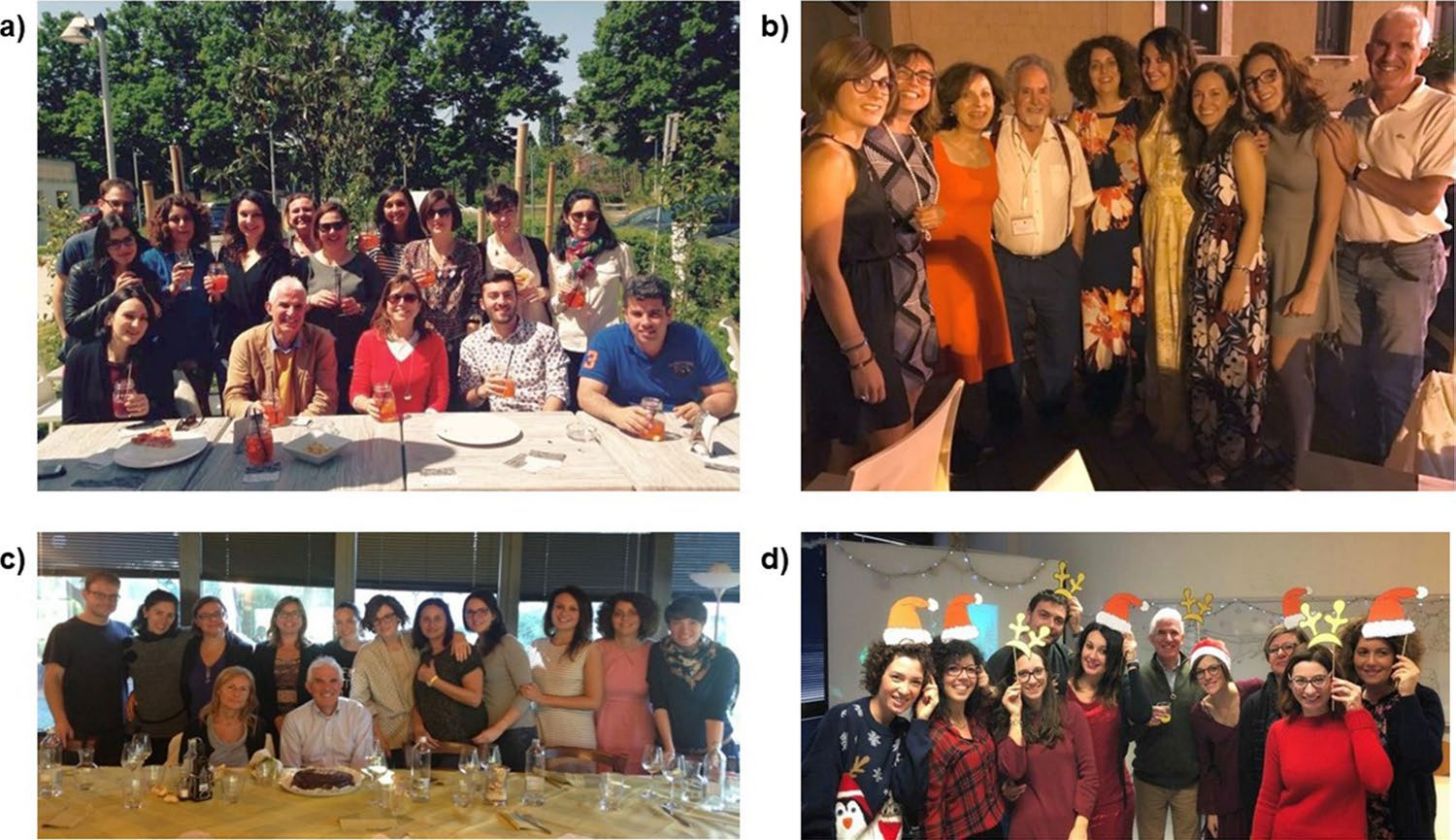
Francesco Di Virgilio. “Group meetings” in **a** 2016, **b** 2017, **c**, **d** 2018. Note the presence of guests Christa Müller, University of Bonn (3rd from left) and Geoffrey Burnstock, University College London (4th from left) in **b**

**Fig. 3:**
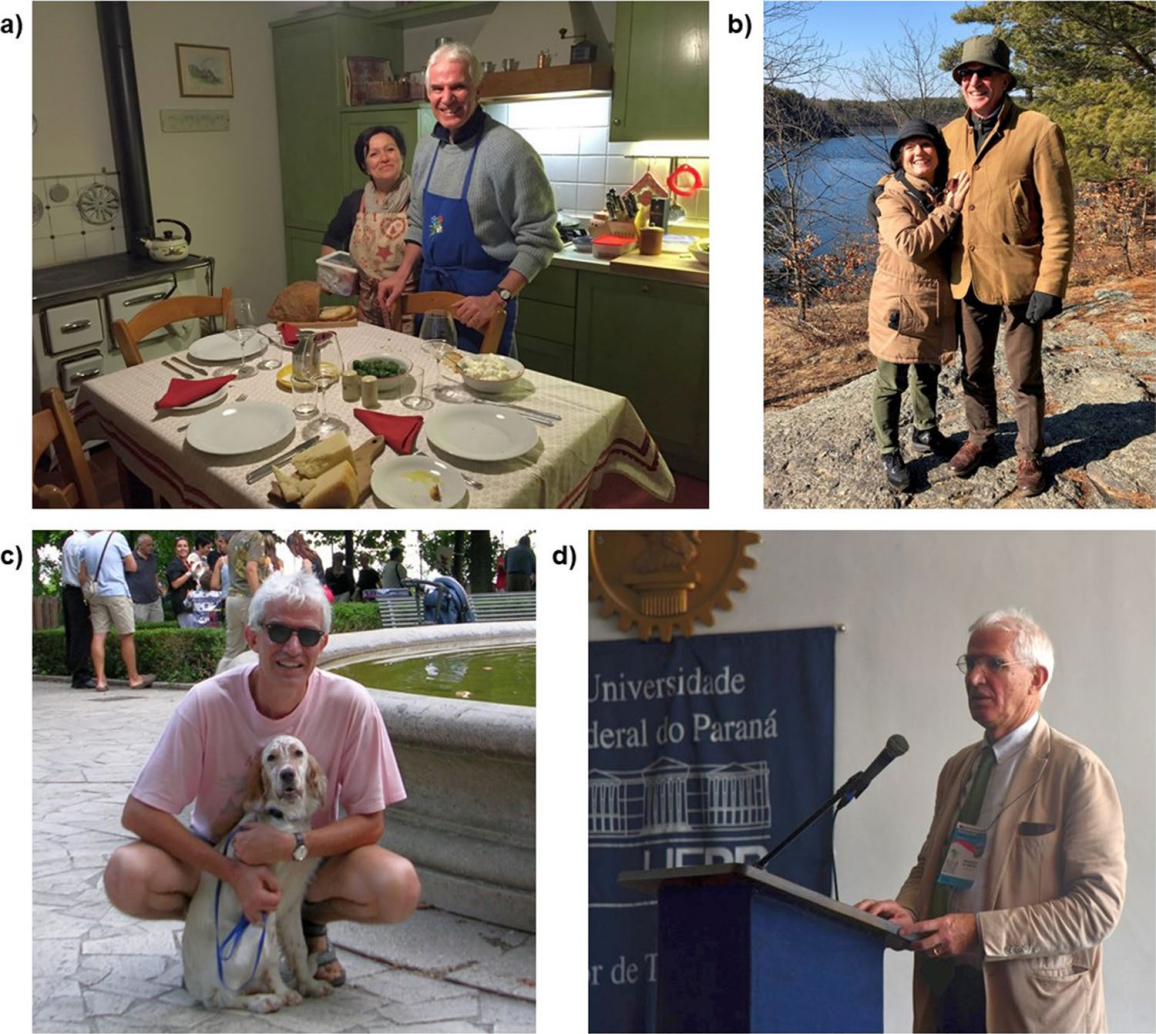
Francesco Di Virgilio. **a**, **b** With Dorianna in 2015 and 2020, respectively. **c** With his dog in 2003. **d** Presenting at the Brazilian Purine Club in Curitiba in 2023

Francesco Di Virgilio, one of the giants of purinergic signalling, died suddenly on September 22, 2024. His passing is an immense loss to so many of us, as friends and colleagues, and especially to his beloved wife, Dorianna. Here, 1 year on, we pay tribute to him and his immense contribution to our field (Figs. 1, 2, and 3).

## Biography

Francesco graduated with a degree in medicine from the University of Padova, Italy, in 1979. His studies and interactions with patients led to what became a lifelong interest in understanding the cellular mechanisms that underlie inflammation. Thus, following compulsory military service in the Italian army as a medical officer, he moved to London in 1982 to work as a research assistant with Bastien Gomperts in the Department of Experimental Pathology at University College London, working on the role of cytoplasmic Ca^2+^ and G proteins in the activation of inflammatory cells [1, 2]. He then returned briefly to the University of Padova as a research assistant in the Laboratory of Pathology and Histopathology in the Institute of General Pathology to study signal transduction in inflammatory cells, before joining Sam Silverstein and Thomas Steinberg in the Department of Physiology and Cellular Biophysics, Columbia University, New York, as a Visiting Fellow Scientist in 1985, to study the molecular mechanisms of phagocytosis. It was during his studies there that he first became acquainted with the actions of ATP at what was then called the P_2Z_-purinoceptor, later to be identified as the P2X7 receptor [3, 4]. He then returned to the University of Padova in 1986 to take up a position as Associate Professor of Molecular Pathology in the Institute of General Pathology, before moving to the University of Ferrara in 1992, where he would spend the rest of his scientific career. Initially, he was an Associate Professor of General Pathology in the Institute of General Pathology, but unsurprisingly, he quickly progressed through the academic ranks and was promoted, first to Professor of General Pathology in the Department of Experimental and Diagnostic Medicine in 1994, then Professor of Clinical Pathology in the Department of Medical Sciences in 2006.

Francesco’s research output was prodigious, publishing over 370 peer-reviewed research papers, reviews, and book chapters, which to date have been cited more than 35,000 times, giving him an H-index above 100. In addition, he filed several patents, including one that described chimeric proteins for measuring ATP concentrations in the pericellular space and the related screening method, another that detailed a method for using phagocytic particles and ATP receptors to deliver antigens to MHC class I receptors to induce immunity against microbial pathogens or tumours, or to suppress immunity, and a third that reported a ratio-metric probe for the measurement of the extracellular ATP concentration. He also collaborated extensively, both within the University of Ferrara and in universities worldwide, including in the UK, Spain, Germany, the USA, and Brazil.

A mark of Francesco’s reputation and standing in science was the large number of important committees and organizations that he was invited to join, such as the IUPHAR Committee for P2X Receptor Nomenclature, the European Academy of Tumor Immunology and Academia Europea, as well as the scientific advisory boards for numerous drug companies. Furthermore, he was a member of the editorial boards of many major scientific journals, including Scientific Reports, the British Journal of Pharmacology, Inflammation, the American Journal of Physiology-Cell Physiology, Signal Transduction and Targeted Therapy, MedComm, Current Opinion in Pharmacology, and Purinergic Signalling. His abilities as a leader were also recognized at the University of Ferrara, and at various times, he served the university as Chairman of the Department of Experimental and Diagnostic Medicine, Dean for Education in the Medical School, Director of the Postgraduate School in Clinical Pathology, Director of the PhD Program in Molecular Medicine and Pharmacology, Deputy Rector for Research and Technology Transfer, and Chairman of the Center of Excellence for the Study of Inflammation, which was established by the Italian Ministry of Education and Scientific Research.

## Personal tributes

### A journey of scientific passion and mentorship

I met Francesco in January 1992, shortly after he had arrived at the University of Ferrara with the goal of establishing a research laboratory focused on purinergic receptors. I was a student looking for a lab to complete the 2 years of formative internship before graduating in Biological Sciences. The early period in his lab was not easy for me because, while Francesco had a great urgency and passion to launch his research line, I had no laboratory experience yet. However, I remember the impressive amount of reading I did, from studies on ion channels and intracellular Ca^2+^ to the discovery of the P2Z receptor.

What struck me most about Francesco was his tenacity, his dedication, and his scientific rigour. Despite his numerous academic commitments, he always found time to personally enter the laboratory, meticulously teaching every procedure and checking the details of each experiment: from the composition of saline solutions to the care of cell cultures. His most quoted phrase was: “Remember, gaining trust and esteem as a scientist takes time, work and sacrifice, while losing it takes just an instant”. Thanks to him, I became passionate about research, and at the end of my internship, he allowed my thesis to be published as my first article [5], also offering the opportunity to present it as a poster at the international conference on adenosine and purinergic receptors in Philadelphia in 1994.

I continued my path with a PhD in experimental pathology under his guidance [6]. At that time, the P2Z receptor was still viewed with scepticism by the academic community, which often frustrated me, but Francesco was used to reassure me with a smile: “Have faith, believe in what you do”. Ferrara, a medieval city with its walls that seem to suffocate the horizon, reflected the social and academic closure that contrasted with his cultural openness. A man of science and classical culture, gifted with an extraordinary memory that ranged from literature to music, Francesco was also a great conversationalist. Coffee breaks in the kitchen with students and collaborators were precious moments during which he ranged with curiosity and passion across anecdotes, historical events, and cultural curiosities, making every encounter a personal enrichment.

Over the years, he has proven to be right: his fame extended far beyond Ferrara, establishing him as a world-renowned scientist and a master in the field of purinergic receptors, particularly the P2Z, renamed as P2X7 receptor, to which he felt deeply connected from the beginning of his career. His contribution has left an unforgettable mark on the international scientific community.

My journey with Francesco, which began in 1992, continued until his passing [7]. I am grateful not only for everything he taught me scientifically, but also for all the moments shared in life. Much of who I am, professionally and culturally, I owe to him. On every birthday and any special occasion, he gifted me a book, a novel, or an essay that he believed could enrich my personal growth. Francesco remains an example of rigour, humanity, and passion, whose scientific and human legacy continues to inspire all those who had the fortune to know him. *Simonetta Falzoni*

### A man of great passion and intuition

I met Francesco and Dorianna for the first time at a meeting in Sicily in 1991. They were not a couple yet but became engaged and married a few years later. At that time, I was working in a different field of research. I started my interest in purinergic signalling in 2007 and joined his research group in 2009 when, in September, I attended a huge meeting on immunity in Berlin, with Francesco and Simonetta Falzoni. Francesco was already very famous thanks to his research on extracellular ATP and the P2X7 receptor, which was becoming a rising star in the inflammation landscape. We worked together until he passed away. Our last publication, a detailed review on the role of purinergic signalling in cancer therapy [8], was published a few days before his death. Francesco had begun studying the role of the P2X7 receptor in in vivo experimental tumour models around 2012, with some pioneering work carried out with Elena Adinolfi and me [9].

Francesco had the ability to see much further than all of us and to understand, with unpredictable intuition, the phenomena we were studying. Thus, research began on the soluble circulating form of the P2X7 receptor, very likely a marker of inflammatory pathologies [10, 11], together with another member of Francesco’s research group, Juana Maria Sanz. Finally, studies on the involvement of the P2X7 receptor in microvesicle release and cellular exchange and in neuroinflammation were carried out during the last years [7].

In addition to research, with Francesco, I also taught medical students. He was passionate about passing on his knowledge in the field of pathology, and he never missed a lecture, unless for very important reasons. We spent a lot of time examining the students together. He was fair in their evaluation and usually the last question in all of the exams was a joke, of course irrelevant to the grade: a geography question. He was indeed fond of geography, as well as of history, literature, music, politics, etc. One could spend hours talking with him about almost anything, always taking home a lot of almost unknown news.

We all miss Francesco, especially at Christmas, when he organized parties in the department, and at birthday parties (Fig. 2), when he always asked for his favourite cake, tiramisù. Francesco’s passing is a great loss for the entire scientific community, as well as for his colleagues, students, friends, and his beloved wife, Dorianna. *Anna Lisa Giuliani*

### A mentor and a role model

I first met Francesco in 1998 during my PhD in Professor Baricordi’s laboratory. From the very beginning, we worked together on the idea that the P2X7 receptor could promote proliferation in immune cells, such as B lymphocytes, particularly those from patients with chronic lymphocytic leukaemia [12]. Later, Francesco introduced me to the group of Anne Marie Surprenant and Alan North at the University of Sheffield, where I completed my PhD and held my first postdoctoral position [13]. Upon returning from England, I began working as a postdoctoral fellow in Francesco’s laboratory until 2011, when I obtained my first permanent position [14–16]. After establishing my own independent laboratory, I continued to collaborate closely with Francesco for many years, focusing on the role of extracellular ATP and the P2X7 receptor in cancer [9, 17, 18].

For me, Francesco was a pivotal figure, both as a mentor and a role model. He taught me scientific rigour and the joy of sharing hypotheses and theories. He was always approachable and kind, with an open door and a genuine enthusiasm for the original ideas of his trainees. He encouraged me to push beyond my limits, both scientifically and personally, supporting my path toward independence and demonstrating the importance of integrity in interactions with colleagues at every level.

Francesco was also a friend with whom I shared many memorable moments, both in Italy and abroad. He was a deeply cultured person, whose reflections were always inspiring, even when far removed from our scientific work. In many ways, he was a true teacher of life. Since his untimely passing, he has been profoundly missed by me and by his many students. I often find myself thinking about how he would have approached certain situations, and I continue to draw inspiration from his memory. I hope that his numerous students and colleagues will carry forward his legacy, and I am certain he would have been pleased to see it endure. *Elena Adinolfi*

### A major scientist and a great friend

I first met Francesco over 30 years ago at a conference in Philadelphia in 1994, and it was the start of a long friendship. As we chatted, we realized that we had both been working at University College London at the same time in the 1980s. I was a PhD student with Geoff Burnstock and he was a post-doctoral fellow with Bastien Gomperts, but we were in different departments, and our paths never crossed. Even before we had met, it was clear to me that Francesco was an outstanding scientist, and this was confirmed and reinforced in the following years. Throughout his career, he addressed the most important questions in his areas of interest. He thought about them deeply and designed experiments that were clearly well-thought-out and rigorously performed. His papers always presented comprehensive data that were presented clearly. Consequently, he made many important discoveries that moved the field forward.

Another important aspect of Francesco was that he was one of the nicest people that you could hope to meet. He was clearly very intelligent, witty with a mischievous sense of humour, and excellent company. Francesco was also an Associate Editor of Purinergic Signalling throughout my time as Editor-in-Chief and a member of the IUPHAR P2X receptor nomenclature sub-committee, of which I am the chair. I very much appreciated his input and commitment to both of these. He helped make the journal the success that it is and played a leading role in producing the most recent definitive review of the properties and functions of P2X receptors [19]. So, we have lost a giant in the field and a dear friend, but he leaves behind an exceptional body of work, an outstanding legacy, and many friends who will miss him. *Charles Kennedy*

### An extensive and productive collaborator

I first met Francesco in 1995 during a preparatory meeting in Magdeburg, organized for German scientists interested in establishing a Collaborative Research Centre of the German Research Council [20]. The planned title of the initiative was “Nucleotides, a new and universal class of extracellular signalling substances”. In addition to the prospective national participants, we also invited a few prominent international scientists to advise us on how best to proceed with the application. One of these was Francesco. We soon discovered a number of shared scientific interests, and during our discussions, we decided to submit a joint application to the Volkswagen Foundation to fund a symposium in Ferrara on “Nucleotides and their receptors in the immune system”. The proposal was promptly approved, and with the support of Pier Andrea Borea and myself, Francesco organized a truly excellent and highly successful meeting [21].

Our paths crossed again in London at a Novartis Foundation Symposium in 2005, where Geoff Burnstock assembled an outstanding programme and invited Francesco to give a talk [22]. As was typical for these symposia, each lecture was followed by lively, in-depth discussions, which were later included in the published book. In the years that followed, we regularly met at gatherings of the purinergic community, including the memorable German–Italian Purine Club meetings, held alternately in cities in Italy and Germany.

I came into particularly close contact with Francesco after joining the faculty of Chengdu University of Traditional Chinese Medicine, China, where I began a productive collaboration with my friend, Yong Tang. Yong had developed an interest in ATP and various purinergic receptors in acupuncture, an idea originally advanced by Geoffrey Burnstock [23]. Inspired by this, we enthusiastically decided to explore how acupuncture-induced purinergic mechanisms might alleviate pain and other pathological conditions [24]. Together, Yong and I founded the International Collaborative Centre on Purinergic Signalling in Chengdu, which we co-directed. It was intended that Francesco would become the next director of the Center, but tragically, he passed away unexpectedly during a symposium in Chengdu in late 2024.

Yong and I cherish particularly warm memories of Francesco from the summer of 2023. Yong visited my wife Patrizia and me at our little house in Lido delle Nazioni at the seaside near to Ferrara, after which we met Francesco in Ferrara and visited his home near Padova. We spent a delightful day exploring the Scrovegni Chapel, admiring Giotto’s magnificent frescoes, and relaxing at the historic Caffè Pedrocchi, chatting animatedly over coffee and ice cream. Francesco and his wife, Dorianna Sandona, graciously hosted us for three nights and we got to know his two beloved dogs, especially his favourite, Isa. I prefer to remember Francesco in these warm and familiar circumstances, surrounded by his family, friends, and dogs, rather than dwell on the sad circumstances of his passing in China. *Peter Illes*

### Our very good friend; a world-class scientist and wonderful colleague

Francesco was the most wonderful, dignified, gifted, and yet very puckish colleague and friend. He was the true Renaissance man, from whom I learnt a great deal about life and science. We spent many pleasant days together at conferences spanning over 20 years, from Woods Hole, MA in the USA, where we first met in person, to Ferrara, Italy in 2024, when he organized the 2nd European Purines Meeting there. This was just days before his last and ill-fated trip to Chengdu.

We had also met and exchanged updates at many other trips to each other’s homes and at multiple other Purine meetings in Spain, Germany, and Brazil, amongst others.

He and his soulmate, Dorianna, graciously hosted me in Ferrara in 2015 (Figs. 1c and 3a, b). This was a most splendid life-affirming time as I became more of a total fan of all things Italian: academically, gastronomically, historically, sporting (soccer), and culturally (Giotto and the Scrovegni Chapel, Padua, and elsewhere). His most recent visit to Boston took place just as COVID had reared its ugly head in Northern Italy for the first time in very early 2020. I recall we hiked in chilly, sunny weather (Fig. 3b) and spoke of shared interests without any real knowledge of what was to happen in the pandemic years and after that. *Carpe Diem*, indeed.

Looking back, he really made great, incisive, and innovative impacts in the area of purinergic signalling. His studies specifically targeted P2X7 receptor function, an area where he was a clear thought leader and the go-to world expert. This scientific role is described so very well above. Francesco’s shared interests with me were in extracellular nucleotide biology and in exploring their roles as mediators of inflammation, innate, and adaptive immunity [25, 26]. In particular, we spoke of the prototypic and sometimes unexpectedly divergent functional activities of CD39 and the P2X7 receptor, in fine-tuning and modulating immunocyte function, cytokine, and chemokine secretion in cancer and chronic disease. He leaves behind a great intellectual legacy that will result in much further work, carried on by Elena Adinolfi and others in Ferrara and elsewhere.

After his 70th birthday, he was looking forward to relaxing in his golden years to grow and harvest grapes and olives with Dorianna and spend time with their hounds, friends, and families. I always thought there were several more decades for Francesco*,* of the good life (goditi la vita); of what Yeats said incomparably in 1899, within “The Song of Wandering Aengus”:

“(to) walk among long dappled grass,/And pluck till time and tides are done,/The silver apples of the moon,/The golden apples of the sun”

This was not to be, and that is really so sad. I will miss him deeply, as will the pursuit of science miss him.

In Genyen’s silent, sacred space,

Near Chengdu, peaks, in high embrace,

We mourn Francesco, gone too soon,

Beneath the calm of monastery moon.

A man of mountains, tall in name,

In science, too, he earned great fame.

With P2X7, his research shone bright,

A clear beacon in the darkest night.

Not just in work but in life, he soared,

With heart and mind in full accord.

And Dorianna, love so true,

Now bears the loss of skies once blue.

High altitudes and sickness came,

To claim a man of great acclaim,

In scaling heights, both near and far,

A true scientist and guiding star.

Though mountains claimed his final breath,

His spirit soars beyond his death.

In peaks and valleys, minds and hearts,

His legacy will ne’er depart.

Francesco, in your quiet rest,

Your life’s great work stands manifest.

Though gone, your light forever burns,

In those whose lives your passion burns.

*Simon C. Robson*

### A scientist with vision and a warm friend

My first contact with Francesco was through a review paper he had written 30 years ago [27]. At that time, I was finishing my PhD and had obtained some results that did not exactly fit with the ideas described by Francesco. Thus, my supervisor, Prof. Pedro Persechini, who had met Francesco some years earlier at the Rockefeller Foundation, decided to write a letter refuting Francesco’s initial writing [28]. I was very nervous and afraid of contradicting a researcher who was already an expert on P2X7 receptors (at that time known as the P2Z receptor) and of entering the purinergic field on the wrong foot. What a surprise! Francesco not only wrote a reply letter, including our hypotheses on the topic, but also invited me to speak at the 6th International Symposium on Adenosine and Adenine Nucleotides, which he was organizing in Ferrara, Italy, in 1998.

This was the beginning of a fruitful professional relationship and a great friendship between our families. At that time, after visiting Francesco’s laboratory, we discussed some projects involving the role of P2Z receptors in protozoan infections. As a result of this initial professional contact, we explored together the involvement of purinergic signalling during *Trypanosoma cruzi* infection and showed that ATP induces an increase in plasma membrane permeabilization and cellular death in CD4 +/CD8 + double-positive thymocytes collected from infected mice during the atrophy phase [29].

In 2004, I invited Francesco to speak at a symposium on “Purinergic signaling during inflammation” at the XIX Annual Meeting of the Federação de Sociedades de Biologia Experimental—FESBE, which took place in Águas de Lindóia, São Paulo, Brazil. It was Francesco and his wife Dorianna’s first trip to South America. It was a great opportunity to discuss science and strengthen social ties. On that occasion, he gave me a picture of his dog (Fig. 3c). After this first visit, Francesco became a friend of Brazilian science. Professors Henning Ulrich and Ana Battastini and I created the Brazilian Purine Club in 2009. Francesco was there and participated in almost every meeting. In 2020, Francesco invited me to consolidate all the ideas we had developed together into a single review [30]. It became one of the most cited reviews in the field, which honours me greatly. I deeply miss this extraordinary scientist and close friend. His contributions to the understanding of P2X7 receptors in the immune system will undoubtedly impact this field for generations to come. *Robson Coutinho-Silva.*

### Gentle and encouraging

I first met Francesco at the 2nd Joint Italian-German Purine Club Meeting, September 2007, in Leipzig, Germany. At that time, I was new in the field and happy to show and discuss my early data. I remember from this meeting that he was very gentle and encouraged me to proceed in the field of purinergic signalling. In 2010, he and Geoffrey Burnstock participated in the first congress of the recently founded Brazilian Purine Club meeting and since then contributed to almost every one of the yearly organized congresses until 2024. He also joined the Brazilian Purine Club Meeting in Curitiba, Brazil; Fig. 3d shows Francesco presenting a plenary lecture and talking to the participants of the congress. We were friends for almost 15 years. Francesco invited me as a speaker in September 2013 at the 5th joint Italian/Brazilian Purine Meeting in his hometown of Ferrara, where I had the honour to give a lecture at his university. I invited Francesco to Brazil in 2024, with the purpose of establishing a collaboration and give a lecture at the yearly congress of the Brazilian Society of Biochemistry and Molecular Biology. I developed a close friendship with Francesco and his wife Dorianna. I met him last at the European Purine Meeting in Ferrara in September 2024, which he chaired. I was deeply sad when I learnt that Francesco passed away a couple of weeks afterwards. His death is an immense loss to the purinergic signalling field to his friends and to me. *Henning Ulrich*

### He will live on in our hearts and in our minds

I first met Francesco in 2019 during the 1 st European Purine Meeting in Santiago de Compostela, Spain. We talked about a future plan to organize an Asian-European Meeting, which was to be held in China, and he made a great number of wonderful suggestions. Due to the COVID-19 pandemic, this meeting could not take place, so we then worked together again to prepare the World Congress on Purines in 2025 [31]. Unfortunately, however, he passed away in 2024 and could not attend this congress. During the organization of the conference, we talked a lot and we agreed that he would be the 2nd Marco Polo after his retirement and this congress, but in purines, and he would come to China to turn his dream into reality because he was one of the first people to work on P2X7 receptors and the structure of P2X7 receptor was solved from the Giant Panda [32], whose hometown is Chengdu, China. During the COVID-19 pandemic, he prepared a couple of online courses for our university to help more graduate and postgraduate students understand basic and cutting-edge knowledge about purines. We also discussed frequently via email the proposal that the P2X7 receptor would be a promising target for the treatment of COVID-19 [33].

In 2023, he came to Chengdu and gave a wonderful talk on “The P2X7 receptor mediates exchange of microparticles and mitochondria by microglial cells” at the 2nd Sino-German Summer School co-organized by Prof. Christian Lohr and me. Peter Illes, Francesco, and I also worked together to organize a special issue on “Purinergic signalling: 50 years” for Neuropharmacology [34]. Then, in 2024, he was invited to give the opening lecture “Extracellular vesicles: a novel information transfer pathway in purinergic signalling” at the 1 st International Conference “Physiology and Pathophysiology of Neuroglia (ICPPN)” co-organized by Prof. Alexei Verkhratsky and me. During this meeting, he was very happy every day with everything, enjoying the science, enjoying talking to his old friends and new friends, and he also planned to bring gifts back to Italy, just like Marco Polo. Throughout this visit to Chengdu I spent so much enjoyable and memorable time with him. He has now left us in the physical world, but he will live on in our heart and in our mind. Many thanks to Francesco for his great contribution to the purine field, especially to the young purine people [35, 36]. I love him very much and will remember him forever. *Yong Tang*

## Data Availability

No datasets were generated or analysed during the current study.
